# Diagnostic Accuracy of Plasma Ghrelin Concentrations in Pediatric Sepsis-Associated Acute Respiratory Distress Syndrome: A Single-Center Cohort Study

**DOI:** 10.3389/fped.2021.664052

**Published:** 2021-05-21

**Authors:** Xiu Yuan, Shaojun Li, Liang Zhou, Tian Tang, Yuwei Cheng, Xiaoxiao Ao, Liping Tan

**Affiliations:** ^1^Department of Emergency, Children's Hospital of Chongqing Medical University, Chongqing, China; ^2^Ministry of Education Key Laboratory of Child Development and Disorders, Chongqing, China; ^3^National Clinical Research Center for Child Health and Disorders (Chongqing), Chongqing, China; ^4^China International Science and Technology Cooperation Base of Child Development and Critical Disorders, Chongqing, China; ^5^Children's Hospital of Chongqing Medical University, Chongqing, China

**Keywords:** sepsis, sepsis-associated PARDS, ghrelin, inflammatory factors, child, respiratory distress syndrome

## Abstract

**Background:** Ghrelin is the endogenous ligand of growth hormone secretagogue receptor 1a, which plays a role in regulating immunity and inflammation. The aim of this study is to assess the diagnostic value of plasma ghrelin in sepsis-associated pediatric acute respiratory distress syndrome (PARDS).

**Methods:** We recruited patients who were admitted to the pediatric ICU (PICU) of the Children's Hospital of Chongqing Medical University between January 2019 and January 2020 and met the diagnostic criteria for sepsis. Data on clinical variables, laboratory indicators, plasma ghrelin concentrations, and inflammatory factors were collected and evaluated, and patients were followed up for 28 days. The area under the receiver-operating characteristic curves (AUROC) were determined using logistic regression to calculate and test cut-off values for ghrelin as a diagnostic indicator of sepsis-associated PARDS. The log-rank test was used to compare survival according to ghrelin levels.

**Main results:** Sixty-six PICU patients (30 with ARDS and 36 without ARDS) who met the diagnostic criteria of sepsis were recruited. The ghrelin level was significantly higher in the ARDS group than in the non-ARDS group. The AUROC of ghrelin was 0.708 (95% confidence interval: 0.584–0.833) and the positivity cutoff value was 445 pg/mL. Sensitivity, specificity, positive predictive value, negative predictive value, positive likelihood ratio, and negative likelihood ratio of plasma ghrelin for the diagnosis of PARDS-associated sepsis were 86.7, 50.0, 59.1, 81.8, 1.734, and 0.266%, respectively. The survival rate of sepsis patients were significantly improved when the ghrelin level was >445 pg/mL.

**Conclusions:** Ghrelin plasma levels were higher in sepsis-associated PARDS, and accompanied by increased levels of inflammatory factors. High ghrelin levels are a positive predictor of ICU survival in sepsis patients. Yet, there is no evidence to prove that elevated ghrelin is a promising diagnostic indicator of sepsis-associated PARDS.

**Trial registration:** Clinicaltrials, ChiCTR1900023254. Registered 1 December 2018 - Retrospectively registered, http://www.clinicaltrials.gov/ChiCTR1900023254.

## Background

Sepsis is defined as a life-threatening organ dysfunction caused by a dysregulated host response to infection, and it is characterized by high incidence, high mortality, and high treatment costs (1). Among all the organs that become vulnerable in sepsis, the lung is considered to be the most vulnerable target organ. Sepsis is often complicated with acute lung injury and even acute respiratory distress syndrome (ARDS), and the latter is one of the main causes of death in sepsis patients (2). Therefore, correct and timely diagnosis and evaluation of sepsis are very important to reduce mortality associated with this condition. Currently, studies have shown the roles of various biomarkers such as cell surface markers, cytokines, procalcitonin (PCT), and C-reactive protein (CRP) in the diagnosis, severity, and prognosis of sepsis (3). Initiation of a systemic inflammatory response forms the basis of the pathogenesis of sepsis-associated pediatric ARDS (PARDS). Some studies have shown that indicators such as tumor necrosis factor-α (TNF-α), interleukin-6 (IL-6), and interleukin-10 (IL-10) are used as early biomarkers of sepsis-associated ARDS (4–6). However, the incidence of ARDS and the mortality factors associated with sepsis-related ARDS are unclear in pediatric patients. Although some progress have been made in research on the pathogenesis and treatment of sepsis-associated ARDS, many recent clinical studies and meta-analyses have demonstrated that the total mortality rate of sepsis-associated PARDS remains as high as 27–45% (7–9).

Ghrelin is an endogenous brain-gut peptide that was first discovered in 1999. It attracted considerable attention at that time, mainly because of its endocrine and appetite regulation functions (10). Ghrelin is an endogenous ligand of the G protein-coupled growth hormone secretagogue receptor (GHSR1a). It is an appetite stimulant and has a regulatory effect on immunity (11). Recent studies have focused on the anti-inflammatory effect of ghrelin. Ghrelin and its receptors are expressed in immune tissues and immune cells that are involved in anti-inflammatory processes and can inhibit the secretion of proinflammatory factors such as IL-6 and TNF-α in activated monocytes, T lymphocytes, and endothelial cells (12). In addition to its anti-inflammatory effects, ghrelin can inhibit apoptosis and improve immunity (13). Recently, clinical studies have demonstrated increased plasma ghrelin concentration in systemic inflammatory models such as severe pancreatitis, acute colitis, inflammatory bowel disease, ankylosing spondylitis, and cystic fibrosis (14). The serum ghrelin concentration has also been reported to increase in critically ill patients (13, 15, 16). However, no studies have investigated the changes in the plasma ghrelin levels in sepsis-associated PARDS. Therefore, in this single PICU cohort study, we explored the correlation between the ghrelin level and sepsis-related ARDS, determined the diagnostic value of ghrelin in sepsis-related PARDS, and analyzed the prognostic value of the plasma ghrelin level in children with sepsis.

## Materials and Methods

### Study Design and Participants

**Figure 1:**
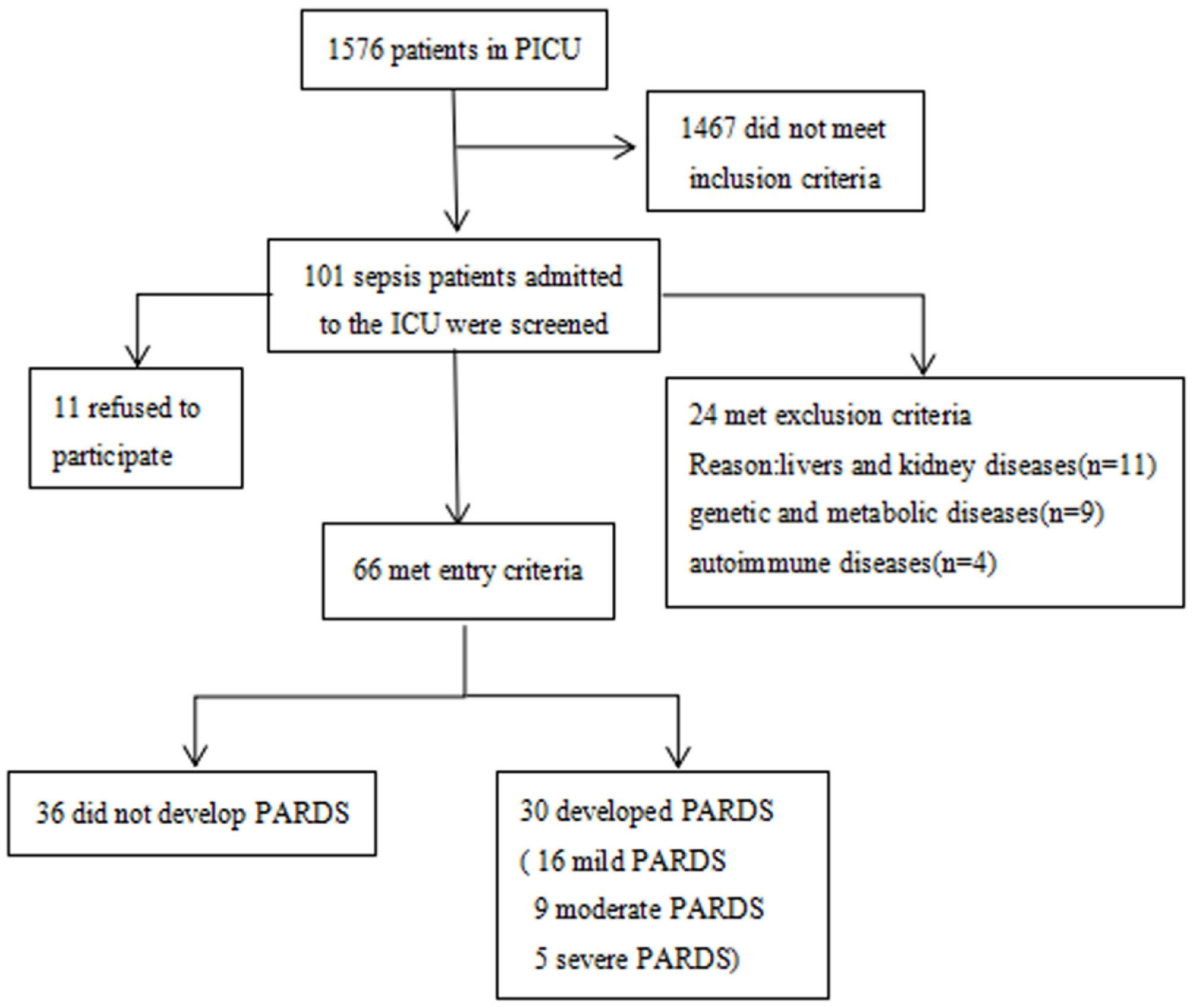
Flowchart of all the patients included in this study.

This study was approved by the local ethics committee (experimental study approval number 271/2019). Written informed consent was obtained from the patient, parent, or designated legal guardian before inclusion in the study. Between January 2019 and January 2020, pediatric patients aged 1 month to 18 years at the PICU of the Children's Hospital affiliated to Chongqing Medical University who met the diagnostic criteria of sepsis (17) were recruited. Patients with underlying diseases such as liver and kidney diseases, genetic and metabolic diseases, and autoimmune diseases, and patients <1 month or older than 19 years of age were excluded from this study (Figure 1).

### Data Collection and Ghrelin Measurement

Data collected included demographic data (sex, age, and body weight), underlying diseases (premature birth, previous lung diseases, neuromuscular disease, and immune deficiency), disease severity variables (pediatric sequential organ failure assessment score [PSOFA], pediatric logistic organ dysfunction [PELOD], pediatric risk of mortality score [PRISM], and septic shock), supportive treatment in the PICU (high-frequency oscillation ventilation [HFOV], extracorporeal membrane oxygenation [ECMO], continuous renal replacement therapy [CRRT], and vasoactive drugs), observation index (glucose concentration, white blood cell count, proportion of neutrophils, and CRP), primary infection (pulmonary infection, intracranial infection, gastrointestinal infection, and bloodstream infection), and outcome variables (hospital days, ICU days, and 28-day mortality). Patients were classified as having sepsis with ARDS if they met the Pediatric Acute Lung Injury Consensus Conference (PALICC) definition of ARDS; patients who did not meet this definition were classified as having sepsis without ARDS (18).

Peripheral venous blood samples of all the patients were obtained after admission to the hospital for diagnosis of sepsis and before therapeutic intervention. After the samples were collected, they were centrifuged within 2 h of collection at 3,000 rpm for 20 min. The plasma-containing supernatant fraction was stored at −80°C until the day of assay. The plasma ghrelin level was investigated using a human ghrelin enzyme-linked immunosorbent assay (ELISA) kit (Upstate Chemicon Linco, Millipore, USA) according to the manufacturer's protocol. Absorbance was read at 450 and 590 nm in a plate reader within 5 min. A reference curve was constructed by plotting the absorbance units at 450 nm subtracted from the absorbance units at 590 nm on the Y-axis against the concentrations of the ghrelin standard on the X-axis. Then, the ghrelin concentration of sepsis patients were measured against this standard curve. Plasma levels of inflammatory molecules IL-6, IL-10, TNF-α, and IL-1β were measured by ELISA according to the manufacturer's instructions (Shen Zhen, NeoBioscience Technology, China). Absorbance was read at 450 nm in a plate reader within 5 min. A reference curve was constructed by plotting the absorbance at 450 nm on the Y-axis against the concentrations of the ghrelin standard on the X-axis. Then, we calculated the IL-6, IL-10, TNF-α, and IL-1β concentrations of patients with sepsis.

### Statistical Analysis

First, we compared the general characteristics of sepsis with or without ARDS. Then, we compared the plasma concentrations of ghrelin and inflammatory factors between sepsis patients with or without ARDS. Next, we studied the correlation between ghrelin and plasma concentrations of these inflammatory factors. Then, we calculated area under the ROC between ghrelin concentration and ARDS, and the positivity cut-off values. The diagnostic test was carried out according to the positivity cut-offs of ghrelin and whether or not sepsis-associated ARDS was present, and the relevant indices such as the sensitivity, specificity, positive predictive value (PPV), negative predictive value (NPV), and Youden index were reported. Positive likelihood ratio (+LR) was calculated as sensitivity (1-specificity), and negative likelihood ratio (–LR) was calculated as (1-sensitivity)/specificity (19). In addition, according to the positivity cut-offs of ghrelin, the patients were divided into two groups, and the survival curve of the two groups were compared.

The sample size calculation for patients were done according to the pre-experimental results, according to which the ghrelin levels in pediatric patients with sepsis with and without ARDS were 403.8 ± 173.8 and 645.9 ± 251.2, respectively. This indicates that a sample size of 16 patients per group is required to obtain a power of 80% for a significance level of 0.05 with a two-tailed test.

Non-normally distributed data were expressed as median and quartile spacing. Two samples were compared using the Wilcoxon rank-sum test, and multiple samples were compared using the Kruskal–Wallis test. Normally distributed data were reported as means ± standard deviation (X ± S). The *t*-test was used to compare the variance between two groups of independent samples. One-way analysis of variance was used to compare the means between multiple samples, and Person's correlation coefficient was used to test the correlation. Correlation analysis was performed using Spearman's correlation coefficient. Logistic regression analysis was used to calculate the area under the ROC between ghrelin concentrations and ARDS, and the positivity cut-offs were calculated. The survival differences between the two groups were compared and analyzed by the log-rank test. SPSS version 21.0 (SPSS, IBM, USA) was used for the statistical analysis.

## Results

### Characteristics of Pediatric Sepsis Patients With or Without ARDS

A total of 101 pediatric patients in the PICU met the diagnostic criteria of sepsis. Of these, 24 were excluded from the study analysis based on the exclusion criteria defined for this study. Finally, 66 patients were enrolled, of which 30 had ARDS and 36 did not have ARDS (Figure 1). The demographic and clinical characteristics of the enrolled patients are shown in Table 1. The quartile range of age in the sepsis with and without ARDS groups were 0.42–1.5 and 0.61–12.17 years, respectively, and no significant differences were found in age and sex between groups (*p* = 2 and *p* = 0.16, respectively). There was no significant difference in underlying diseases, disease severity, and supportive treatment in the PICU during hospitalization between the two groups. The difference in primary infection at enrollment was significant between the two groups (*p* = 0.001). Pulmonary infection was the main infection in the sepsis group with ARDS. In the 28-day follow-up, 9 of the 30 patients in the sepsis with ARDS group died and 10 of the 36 patients in the sepsis without ARDS group died, but the difference between the two groups were not statistically significant (*p* = 0.843). Other patient outcomes (such as PICU hospitalization time and ICU days) significantly differed between the sepsis with ARDS and sepsis without ARDS groups (*p* = 0.03, *p* < 0.001).

**Table 1:** Baseline patient characteristics.

**Parameter**	**All patients (*n* = 66)**	**Sepsis without ARDS (*n* = 36)**	**Sepsis with ARDS (*n* = 30)**	***P*-value**
**Baseline demographic**				
Sex (male/female)	37/29	23/13	14/16	0.16
Age median [quartile (month)]	9.84 (3.84-63.72)	27.96 (7.32–146.04)	8.64 (5.04–18)	0.2
Body weight median [quartile (kg)]	10 (8–28.12)	12.25 (9.55–35.75)	8 (6–21)	0.058
**Underlying diseases [*****n*** **(%)]**				
All underlying diseases	15 (22.7%)	5 (13.9%)	10 (33.3%)	0.064
Premature birth	3 (4.5%)	0 (0.0%)	3 (10%)	0.117
Previous lung diseases	9 (13.6%)	5 (13.9%)	4 (13.3%)	0.948
Neuromuscular disease	2 (3.0%)	0 (0.0%)	2 (6.7%)	0.394
Immune deficiency	2 (3.0%)	0 (0.0%)	2 (6.7%)	0.203
**Disease severity**				
PRISM III	7 (3–12)	11 (6–16.25)	6 (3–9)	0.333
PELOD−2	5 (2–8)	7.5 (3.25–9)	5 (2–7)	0.577
pSOFA Scoring	4.5 (3–7)	5 (4–8)	5 (3–7)	0.378
Septic shock	37 (56.1%)	24 (66.7%)	13 (43.3%)	0.057
**Supportive treatment of PICU during hospitalization [*****n*** **(%)]**				
HFOV	1 (1.6%)	0 (0.0%)	1 (3.3%)	0.476
ECMO	2 (3.2%)	0 (0.0%)	2 (6.7%)	0.223
CRRT	5 (7.9%)	1 (3.0%)	4 (13.3%)	0.296
Vasoactive drugs	35 (55.6%)	13 (39.4%)	22 (73.3%)	0.007
**Observation index**				
Glucose concentration [median (quartile)]	5.95 (4.9–7.3)	6.3 (4.45–8.2)	6.6 (5.4–7.5)	0.597
White blood cell count [median (quartile)]	9.54 (6.63–14.93)	9.82 (6.33–16.25)	8.81 (5.26–22.48)	0.634
Proportion of neutrophils [median (quartile)]	0.72 (0.59–0.84)	0.73 (0.65–0.84)	0.71 (0.49–0.84)	0.529
C-reactive protein [median (quartile)]	29.5 (9.75–65.25)	10 (5.25–54.75)	31 (9–91)	0.747
**Detection index**				
Plasma ghrelin median [quartile (pg/ml)]	598.37 (408.86–1153.0)	506.75 (297.46–1341.45)	645.01 (500.06–1147.6)	0.004
Plasma IL−6 median [quartile (pg/ml)]	100.72 (60.88–177.92)	80.74 (50.57–151.88)	114.71 (69.93–284.75)	0.015
Plasma TNF-α median [quartile (pg/ml)]	14.87 (7.33–25.97)	12.44 (2.94–17.65)	14.87 (8.06–35.46)	0.012
Plasma IL-10 median [quartile (pg/ml)]	11.73 (4.31–26.55)	11.97 (3.77–24.92)	12.76 (5.92–26.93)	0.057
Plasma IL-1β median [quartile (pg/ml)]	8.96 (4.11–33.62)	4.78 (3.53–10.59)	19.82 (7.76–141.50)	0.005
**Primary infection [*****n*** **(%)]**				0.001
Pulmonary infection	*n =* 30 (45.4%)	*n =* 11 (30.5%)	*n =* 19 (63.3%)	–
Intracranial infection	*n =* 7 (10.6%)	*n =* 7 (19.4%)	*n =* 0 (0%)	–
Gastrointestinal infection	*n =* 21 (1.8%)	*n =* 16 (44.4%)	*n =* 5 (16.6%)	–
Bloodstream infection	*n =* 2 (3%)	*n =* 0 (0%)	*n =* 2 (6.6%)	–
Other infection	*n =* 6 (9%)	*n =* 2 (5.4%)	*n =* 4 (12.12%)	–
**Outcome index**				
Hospital days [median (quartile)]	14.5 (8–23)	14.5 (3.25–20)	19 (15–31)	0.03
ICU days [median (quartile)]	6.5 (3–12)	6 (2–9.5)	10 (7–15)	0.000
28–day mortality [*n* (%)]	19 (28.8%)	10 (27.8%)	9 (30%)	0.843

*Data are expressed as n (%) or median (IQR) unless otherwise stated. p-values are calculated with the χ2, Fisher's exact, or Mann-Whitney U-test for differences between the sepsis with ARDS and sepsis without ARDS groups. PRISM III, Pediatric risk of mortality III; PELOD-2, Pediatric Logistic Organ Dysfunction 2; SOFA, sepsis-related organ failure assessment; HFOV, high-frequency oscillation ventilation; ECMO, extracorporeal membrane oxygenation; CRRT, continuous renal replacement therapy; IL-6, interleukin-6; TNF- α, tumor necrosis factor-α; IL-10, interleukin-10; IL-1β, interleukin-1β*.

### Analysis of Ghrelin Plasma Levels

**Figure 2:**
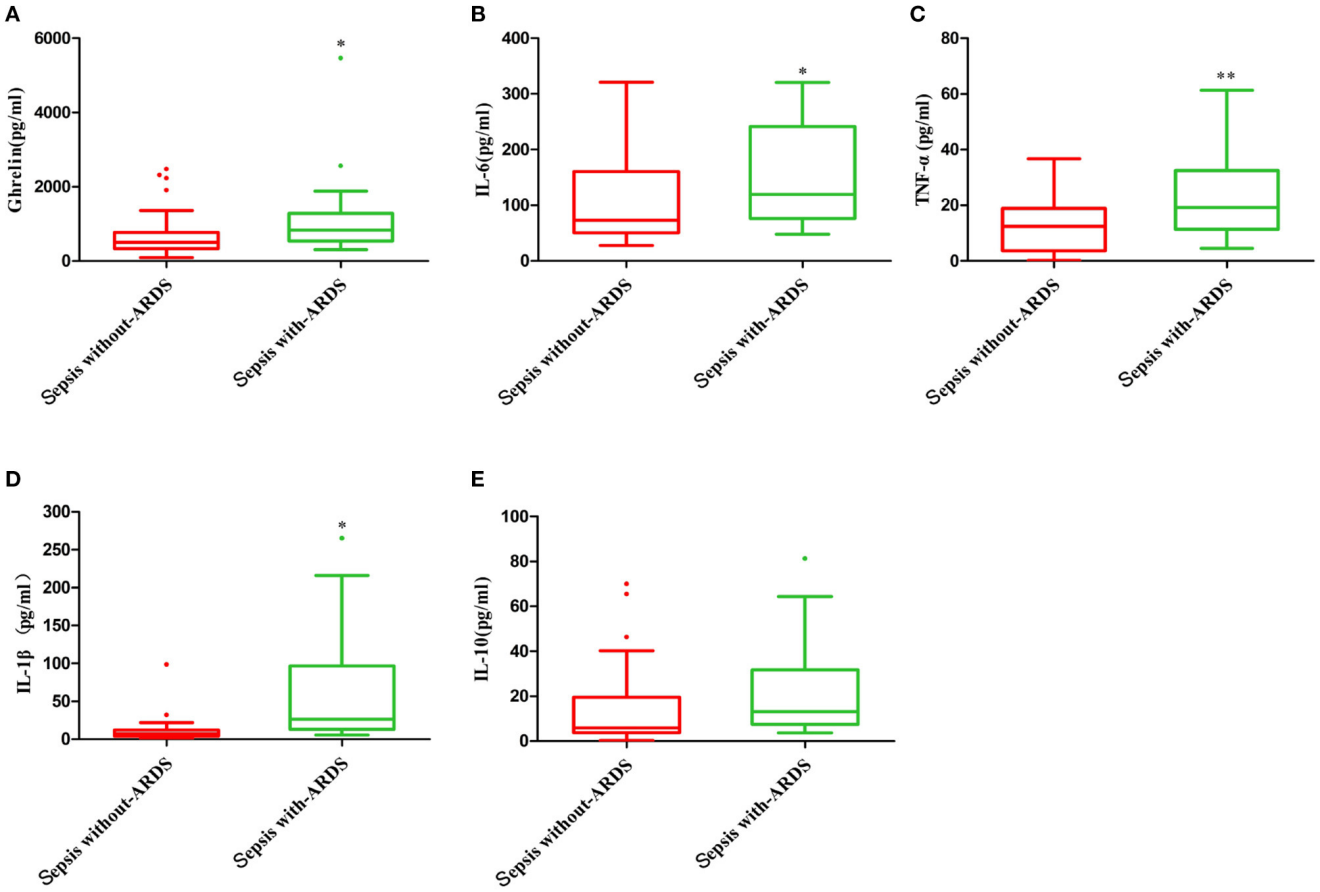
Plasma ghrelin level and concentration of inflammatory factors in sepsis with and without ARDS. **(A)** ghrelin, **(B)** IL-6, **(C)** TNF-α, **(D)** IL-1β, and **(E)** IL-10. *p < 0.05; **p < 0.001.

The plasma ghrelin level was significantly higher in the sepsis with ARDS group than in the sepsis without ARDS group (*p* = 0.048). The levels of inflammatory cytokines IL-6, TNF-α, and IL-1β in the sepsis with ARDS group were all significantly higher than that in the sepsis without ARDS group (*p* = 0.049, *p* = 0.006, *p* = 0.048, respectively), while the IL-10 level in sepsis patients with ARDS did not significantly differ from that in sepsis patients without ARDS (*p* = 0.106; Figure 2).

**Figure 3:**
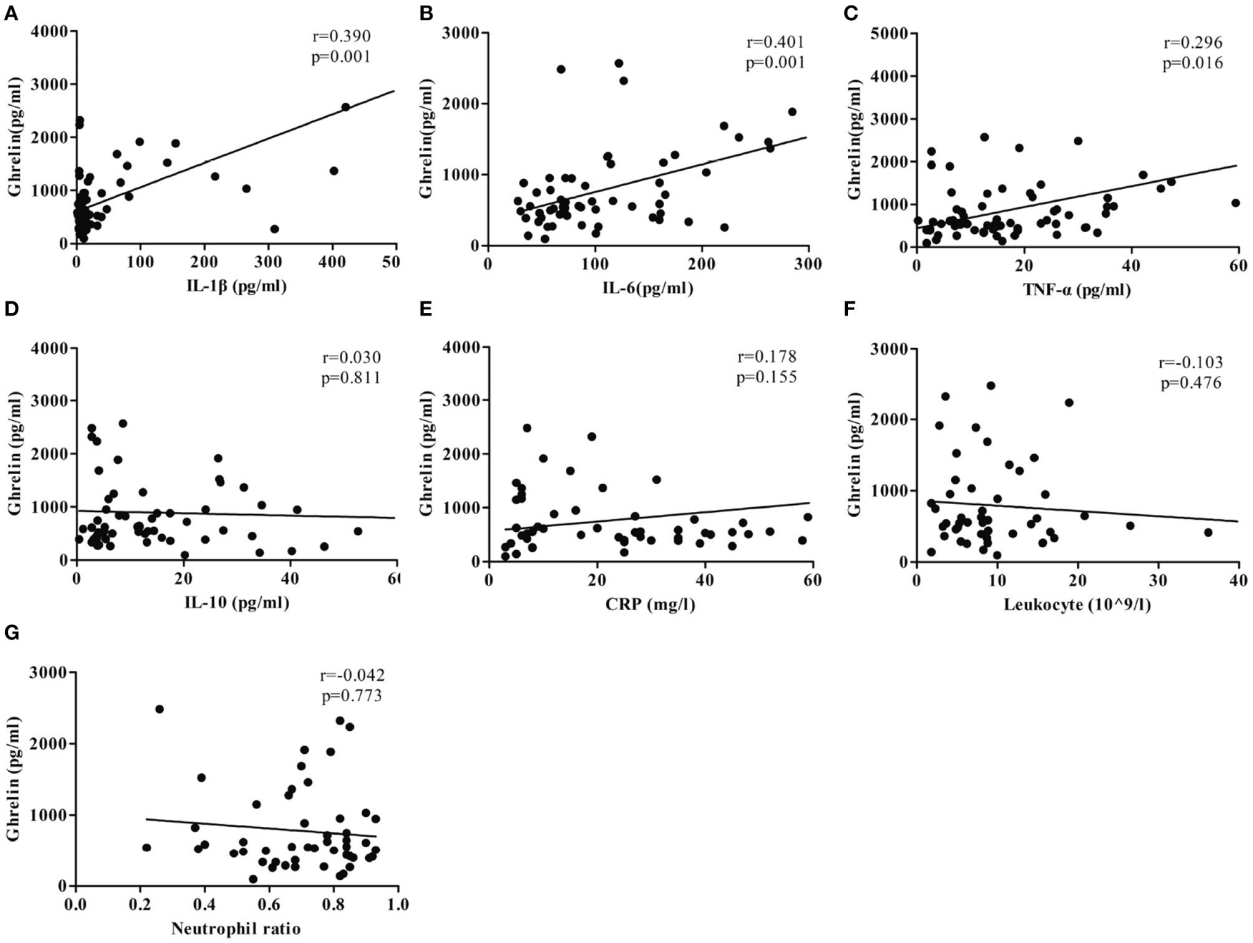
Correlation between the ghrelin level and plasma concentrations of inflammatory factors. **(A)** IL-1β. **(B)** IL-6. **(C)** TNF-α. **(D)** IL-10. **(E)** CRP. **(F)** Leukocyte (109/L). **(G)** Neutrophil ratio.

The plasma ghrelin level was positively correlated with the IL-6, TNF-α, and IL-1β levels (*r* = 0.401, *p* = 0.001; *r* = 0.296, *p* = 0.015; and *r* = 0.390, *p* = 0.001; respectively), while it was not correlated with the CRP, leukocyte, and neutrophil counts (*r* = 0.178, *p* = 0.155; *r* = −0.122, *p* = 0.397; *r* = −0.101, *p* = 0.485; respectively; Figure 3).

**Figure 4:**
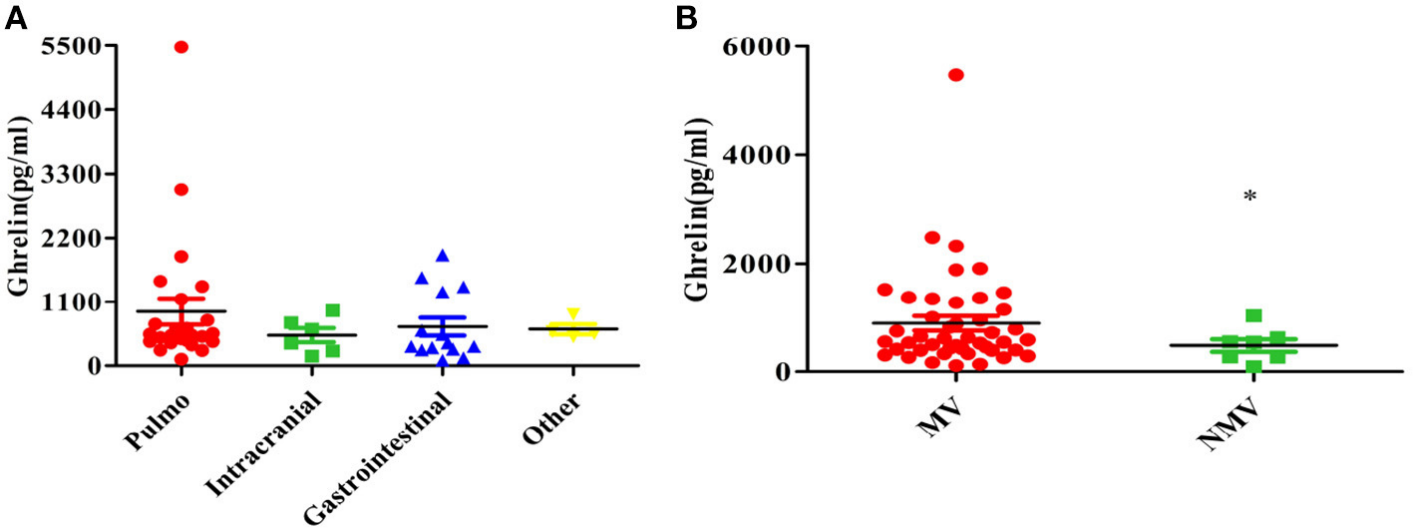
Relationship between the plasma ghrelin level and other clinical factors in patients with sepsis. **(A)** Primary infection. **(B)** Mechanical ventilation. *p < 0.01. MV, mechanical ventilation; NMV, no mechanical ventilation.

Pulmonary infection was the primary infection in the patients, and other primary infections included intracranial, gastrointestinal, and blood infections (Table 1). There was no significant difference in the plasma ghrelin level according to primary infection (*p* = 0.550; Figure 4A).

Our study found that the ghrelin levels were high in sepsis patients with mechanical ventilation than in those without mechanical ventilation (*P* = 0.010; Figure 4B).

### Diagnostic and Prognostic Efficacy of Plasma Ghrelin

**Figure 5:**
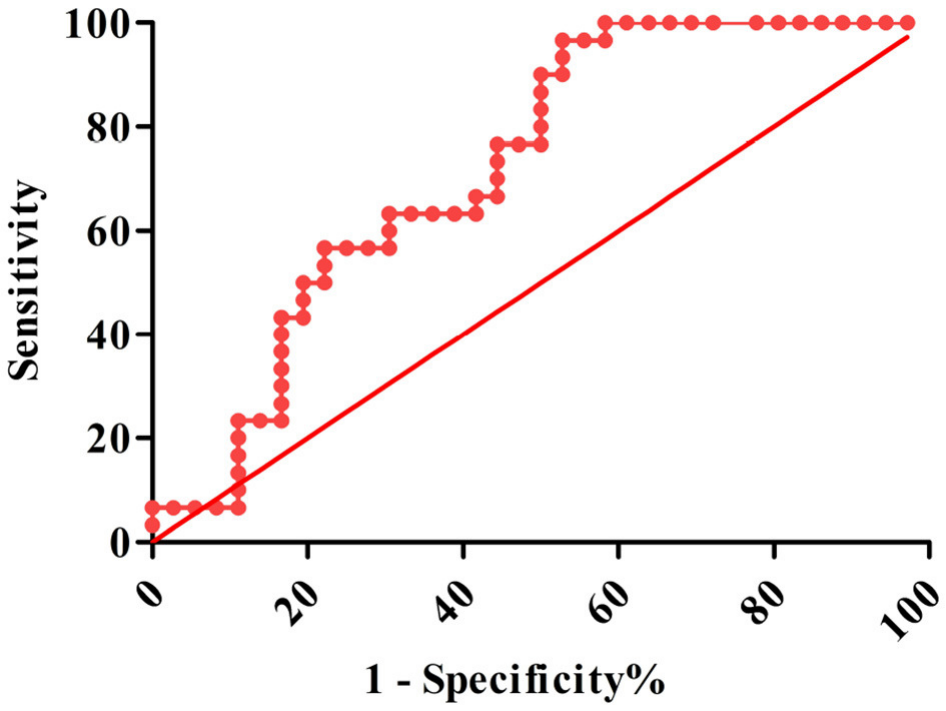
ROC of plasm ghrelin level for diagnosis ARDS in sepsis.

The AUROC for ghrelin was 0.708 (95% CI: 0.584–0.833, *P* < 0.001), and the optimal cutoff value was 445 pg/ml. When the ghrelin value was 445 pg/ml, the sensitivity and specificity of the plasma ghrelin level for predicting sepsis with ARDS were 0.867 and 0.500, respectively, and the PPV, NPV, +LR, –LR, and Yuden index of ghrelin for the diagnosis of sepsis with ARDS were 59.1, 81.8, 1.734, 0.266, and 0.367%, respectively (Table 2 and Figure 5).

**Table 2:** Diagnostic evaluation index of plasm ghrelin.

**Index**	**Ghrelin**
AUC(95% Cl)	0.708(0.584–0.833)
Cutoff values	445 pg/ml
Sensitivity	0.867
Specificity	0.500
PPV	0.591
NPV	0.818
+LR	1.734
–LR	0.266
Yuden index	0.367

*OR, odds ratio; Cl, Confidence level; AUC, area under the curve; PPV, positive predictive value; NPV, negative predictive value; +LR, positive likelihood ratio; -LR, negative likelihood ratio*.

**Figure 6:**
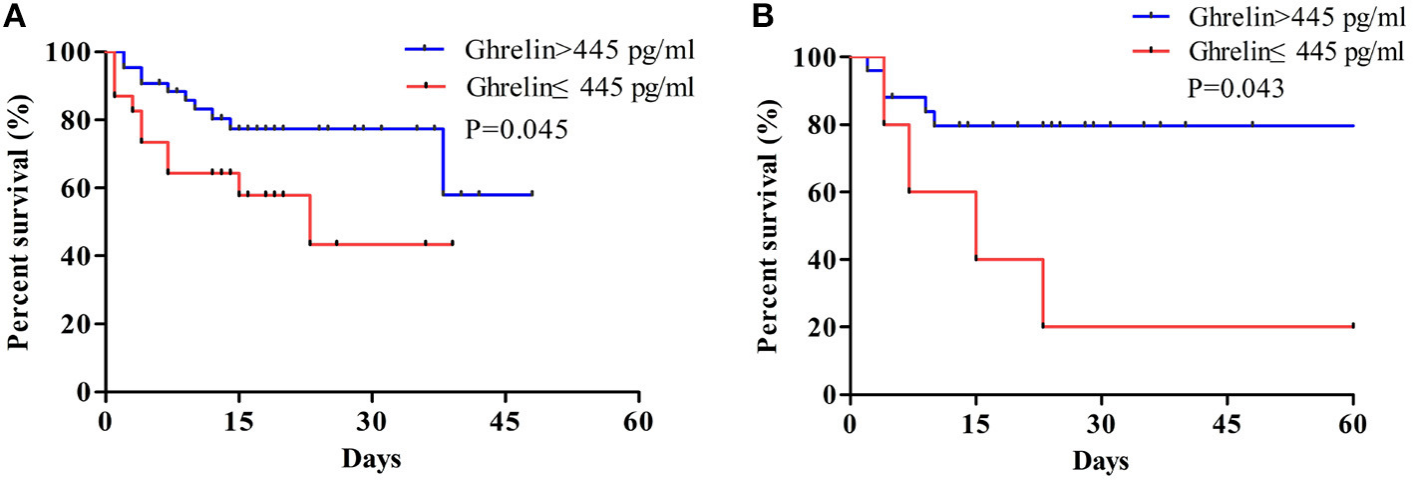
**(A)** Survival analysis of plasma ghrelin level for sepsis. **(B)** Survival analysis of plasma ghrelin level in sepsis with ARDS patients. The log-rank test was used to test the difference in survival rate between groups where the plasma ghrelin value was ≥445 and <445 pg/ml.

The results of the survival analysis showed that high ghrelin plasma concentration was a good predictor of favorable prognosis in patients with sepsis. As shown in the curve, the 28-day survival rate of patients with sepsis were significantly improved when the ghrelin level was >445 pg/mL than when it was <445 pg/mL (*P* = 0.040; Figure 6).

## Discussion

Globally, sepsis is a serious problem in children, and is associated with high mortality and economic burden, and the fatality rate further increases in patients with sepsis who have ARDS (20). The SOFA, PELOD, and PRISM scores can be used to judge the severity and prognosis of the disease, including sepsis, but its value in predicting ARDS is limited; therefore, developing reliable biomarkers can improve the early identification of ARDS and provide a reference for early intervention and the development of new treatments (21–23). We conducted a ghrelin diagnostic test for pediatric sepsis patients with ARDS. The ROC curve of ghrelin revealed that the AUC of sepsis with ARDS was 0.708, with moderate discrimination. According to the best cutoff value of ghrelin, authenticity evaluation showed good sensitivity and poor specificity, satisfactory NPV and acceptable PPV, and acceptable –LR and +LR. Therefore, there is no evidence to show that ghrelin may be used as a biomarker for the early prediction of ARDS in sepsis patients.

Ghrelin is an endogenous ligand for GHSR-1a, and its primary function is to regulate inflammation and immunity (24–27). In an animal model of sepsis, ghrelin was shown to improve tissue hypoperfusion in severe sepsis (28). Another clinical study reported that the plasma ghrelin concentration was significantly increased in adult patients with sepsis, and also suggested that ghrelin may be protective in septic patients (29). Previous animal studies in animal models of hemorrhagic shock have found that ghrelin might be a protective factor in lung injury (30). In a model of LPS-induced rat alveolar macrophage (AM) sepsis, ghrelin was found to protect AMs against apoptosis *in vivo* and *in vitro* through GHSR-1a, which helps reduce sepsis-induced ARDS (31). To the best of our knowledge, there is no scientific report on the protective effect of ghrelin in lung injury. Our results showed that the plasma ghrelin level was significantly higher in children with sepsis with ARDS than in those with sepsis without ARDS. Moreover, we found that the IL-6, TNF- α, and IL-1β concentrations remained consistent. Further analysis showed that the ghrelin level was positively correlated with the CRP, IL-6, TNF-α, and IL-1β concentrations. The results showed that the more serious the condition, the higher was the degree of inflammation and the level of IL-6, TNF-α, and CRP, as well as ghrelin. These findings indicate that the increase in ghrelin level in critically ill patients may be attributable to the anti-inflammatory response of the body.

The study have found that ghrelin administration improves intestinal barrier dysfunction caused by sepsis in animal models, which have shown that the cause of abdominal sepsis is associated with ghrelin levels (32). Some studies have reported that the level of ghrelin is lower in patients with advanced liver cirrhosis than in patients with mild liver cirrhosis under inflammatory conditions; further, ghrelin may be involved in the regulation of autophagy to prevent cell damage in patients with hepatitis (33, 34). So to test the impact of the underlying etiology of critical illness we performed extensive subgroup analysis. The present findings showed that the level of plasma ghrelin was slightly higher in children with a primary pulmonary infection than in children with other infection sites, but the difference was not statistically significant. Thus, it is difficult to make a clear conclusion about the role of ghrelin in the lung based on the present findings and further investigation along this line is warranted.

Studies have shown that ghrelin plays a protective role in septic lung injury by reducing the production of pro-inflammatory factors IL-1β, TNF-α, and IL-6 from AMs via inhibition of the NF-κB/iNOS pathway or Akt signaling (35). Mechanical ventilation is usually administered in patients with severe lung disease and lung dysfunction. Accordingly, in the present cohort, the ghrelin levels were higher in patients who were on mechanical ventilation than in those who were not on mechanical ventilation. In addition, Studies from animal models have demonstrated that ghrelin injection into rats with sepsis led to an increase in the ghrelin level in their lungs, alleviation of lung injury, increase in the pulmonary blood flow, downregulation of proinflammatory cytokines, inhibition of NF-kB activation, and increased survival rate (36). The results of an adult critical illness/sepsis cohort study showed that sepsis patients with higher ghrelin levels had better survival (13, 37). There are no relevant studies among pediatric patients with sepsis, therefore, we analyzed the survival rates of pediatric sepsis patients and sepsis with ARDS with different plasma ghrelin concentrations. Our study found that the survival rate of patients were higher when their plasma ghrelin concentration was >445 pg/mL than when their plasma ghrelin concentration was <445 pg/ml. Therefore, ghrelin may be a positive predictor of sepsis in both children and adults. For patients with sepsis, ghrelin levels were higher in mechanically ventilated patients than those in non-mechanically ventilated patients, and higher ghrelin levels were associated with high survival rate in patients of sepsis with ARDS. Our diagnostic tests showed that ghrelin is fair sensitive to sepsis with ARDS. Therefore, we speculated that ghrelin may play a protective role in sepsis-mediated lung injury through the action of anti-inflammatory cytokines. However, our outcomes showed that the increased ghrelin levels were associated with decreased mortality in patients with sepsis, which may result in poor specificity of ghrelin as a diagnostic test. Because of poor specificity of ghrelin, our results are not sufficient to evidence that ghrelin can be used as a biomarker for early prediction of ARDS in patients with sepsis.

This is the first study to verify the diagnostic value of ghrelin in children with sepsis-associated ARDS, and explore the correlation between ghrelin levels and sepsis with ARDS in children with sepsis. Furthermore, this study analyzed the prognostic value of the plasma ghrelin level in children with sepsis. There are some limitations in this study. First, this is a single-center PICU cohort of children with sepsis, so it is difficult to extrapolate the findings to other populations. Second, There is little difference between body weights and underlying diseases. Lastly, this study did not explore other biomarkers with the potential to have a joint diagnostic value in combination with ghrelin.

## Conclusion

Our study suggests that ghrelin may be a protective factor for lung injury in children with sepsis. However, there is no evidence to show that the ghrelin level may be a promising diagnostic indicator of sepsis with PARDS. In addition, the ghrelin levels may be a positive predictor of sepsis.

## Data Availability Statement

The original contributions presented in the study are included in the article/supplementary material, further inquiries can be directed to the corresponding author/s.

## Ethics Statement

The studies involving human participants were reviewed and approved by the Ethics Committee of Children's Hospital of Chongqing Medical University. Written informed consent from the participants' legal guardian/next of kin was not required to participate in this study in accordance with the national legislation and the institutional requirements. Written informed consent was not obtained from the minor(s)' legal guardian/next of kin for the publication of any potentially identifiable images or data included in this article.

## Author Contributions

LT designed the study. XY performed study selection, assessment of validity, data analysis, and writing of the paper. SL, LZ, TT, YC, and XA participated in the revision of the paper. LZ, TT, YC, and XA performed data extraction and retrieval of bibliographies. All authors were responsible for the content of this manuscript and approved to submit.

## Conflict of Interest

The authors declare that the research was conducted in the absence of any commercial or financial relationships that could be construed as a potential conflict of interest.

## Abbreviations

PARDS: Pediatric acute respiratory distress syndrome
GHSR1a: Growth hormone secretagogue receptor 1a
PPV: Positive predictive value
NPV: Negative predictive value
+LR: Positive likelihood ratio
–LR: Negative likelihood ratio
PCT: Procalcitonin
CRP: C-reactive protein
TNF- α: Tumor necrosis factor-α
IL-6: Interleukin-6
IL-10: Interleukin-10
PSOFA: Pediatric sequential organ failure assessment score
PELOD: Pediatric logistic organ dysfunction
PRISM: Pediatric risk of mortality score
PALICC: Pediatric Acute Lung Injury Consensus Conference
ELISA: Enzyme-linked immunosorbent assay
AM: Alveolar macrophage.
